# A structured approach to applying systems analysis methods for examining implementation mechanisms

**DOI:** 10.1186/s43058-023-00504-5

**Published:** 2023-10-19

**Authors:** Bo Kim, Gracelyn Cruden, Erika L. Crable, Andrew Quanbeck, Brian S. Mittman, Anjuli D. Wagner

**Affiliations:** 1 VA Boston Healthcare System, 150 South Huntington Avenue, Boston, MA 02130 USA; 2 Harvard Medical School, 25 Shattuck Street, Boston, MA 02115 USA; 3 Chestnut Health Systems, Lighthouse Institute-Oregon Group, 1255 Pearl Street, Eugene, OR 97401 USA; 4 UC San Diego, 9500 Gilman Drive, La Jolla, CA 92093 USA; 5 Child and Adolescent Services Research Center, 3665 Kearny Villa Road, San Diego, CA 92123 USA; 6 UC San Diego ACTRI Dissemination and Implementation Science Center, 9500 Gilman Drive, La Jolla, CA 92093 USA; 7 University of Wisconsin-Madison, 610 North Whitney Way, Madison, WI 53705 USA; 8 Kaiser Permanente Southern California, 200 North Lewis Street, Orange, CA 92868 USA; 9 University of Southern California, 2025 Zonal Avenue, Los Angeles, CA 90089 USA; 10 UCLA, 405 Hilgard Avenue, Los Angeles, CA 90095 USA; 11 University of Washington, 3980 15Th Avenue NE, Seattle, WA 98195 USA

**Keywords:** Implementation mechanisms, Implementation strategies, Health care systems, Systems engineering, Systems science

## Abstract

**Background:**

It is challenging to identify and understand the specific mechanisms through which an implementation strategy affects implementation outcomes, as implementation happens in the context of complex, multi-level systems. These systems and the mechanisms within each level have their own dynamic environments that change frequently. For instance, sequencing may matter in that a mechanism may only be activated indirectly by a strategy through another mechanism. The dosage or strength of a mechanism may vary over time or across different health care system levels. To elucidate the mechanisms relevant to successful implementation amidst this complexity, systems analysis methods are needed to model and manage complexity.

**Methods:**

The fields of systems engineering and systems science offer methods—which we refer to as systems analysis methods—to help explain the interdependent relationships between and within systems, as well as dynamic changes to systems over time. When applied to studying implementation mechanisms, systems analysis methods can help (i) better identify and manage unknown conditions that may or may not activate mechanisms (both expected mechanisms targeted by a strategy and unexpected mechanisms that the methods help detect) and (ii) flexibly guide strategy adaptations to address contextual influences that emerge after the strategy is selected and used.

**Results:**

In this paper, we delineate a structured approach to applying systems analysis methods for examining implementation mechanisms. The approach includes explicit steps for selecting, tailoring, and evaluating an implementation strategy regarding the mechanisms that the strategy is initially hypothesized to activate, as well as additional mechanisms that are identified through the steps. We illustrate the approach using a case example. We then discuss the strengths and limitations of this approach, as well as when these steps might be most appropriate, and suggest work to further the contributions of systems analysis methods to implementation mechanisms research.

**Conclusions:**

Our approach to applying systems analysis methods can encourage more mechanisms research efforts to consider these methods and in turn fuel both (i) rigorous comparisons of these methods to alternative mechanisms research approaches and (ii) an active discourse across the field to better delineate when these methods are appropriate for advancing mechanisms-related knowledge.

**Supplementary Information:**

The online version contains supplementary material available at 10.1186/s43058-023-00504-5.

Contributions to the literature

- We offer a four-step approach to applying systems analysis methods for identifying, specifying, testing, and refining the understanding of implementation mechanisms that need to be activated for implementation strategies to lead to desirable implementation, service, and clinical outcomes.
- Systems analysis methods can capture and reflect synergistic, antagonistic, or other non-additive patterns of co-occurrence, especially for multiple strategies that target distinct mechanisms and are thus bundled to implement interventions into multi-level systems (which are common in health care and community settings).
- Knowledge of such patterns is particularly crucial for implementation that must carefully plan how to allocate resources across different strategies.

## Background

The field of implementation science pursues knowledge regarding methods that successfully promote the uptake of evidence-based interventions into routine practice [1]. Such methods tested by the field are implementation strategies, which are “techniques used to enhance the adoption, implementation, and sustainability” of an intervention [2]. An implementation strategy’s success often varies across different implementation contexts [3]—i.e., the success of an implementation strategy is influenced by who uses the strategy, for what purpose it is employed, and also when, where, and how the strategy is used. There is a growing interest in understanding the specific mechanisms through which an implementation strategy functions to achieve optimal implementation outcomes [4]. In other words, enhancing our knowledge of mechanisms is critical to learning why a specific implementation strategy works or not in moving evidence into practice within a given context. Failure to learn why implementation strategies work may result in the use of sub-optimal strategies that do not activate the desired process or inadvertently activate unplanned processes, leading to unintended and undesirable implementation and health outcomes.

Lewis and colleagues [4] define an implementation mechanism as a “process or event through which an implementation strategy operates to affect desired implementation outcomes.” Mechanisms include moderators (factors that affect the strength and direction of a relationship) and mediators (factors that sit between the strategy and the outcome and can account for an observed relationship between the two). Notably, not all mediators will serve as mechanisms. Table 1 summarizes the implementation mechanism-related terms as defined by Lewis and colleagues.

**Table 1:** Implementation mechanism-related terms and definitions (adapted from [4])

Term	Definition
Precondition	Factor that is necessary in order for an implementation mechanism to be activated
Mechanism	Process or event through which an implementation strategy operates to affect desired implementation outcomes
Mediator	Intervening variable that may account for the relationship between the implementation strategy and the implementation outcome (Note: although mechanisms are always mediators, not all mediators are mechanisms)
Moderator	Factor that increases or decreases the level of influence of an implementation strategy
Proximal outcome	Product of the implementation strategy that is realized because of its specific mechanism of action; the most immediate, observable outcome in the causal pathway
Distal outcome	What the implementation process is ultimately intended to achieve

In Lewis and colleagues’ seminal article, they describe how a “training” implementation strategy can increase clinicians’ use of an evidence-based intervention because it works through the “skill building” mechanism. In this example (which we will refer to and further explain under the “Steps to apply systems analysis methods for studying implementation mechanisms” section), this mechanism is meant to increase the clinicians’ use of the focal evidence-based practice. When a chosen implementation strategy does not achieve the desired implementation outcomes (i.e., clinicians’ use does not increase), it could be that (i) appropriate preconditions were not met for the mechanism to take place (or “be activated”; e.g., clinicians’ work schedules did not allow them to attend training sessions), (ii) preconditions were met but other factors attenuated the strategy’s impact (e.g., clinicians’ low desire to learn), or (iii) there were additional variables along the causal pathway that hindered the strategy’s impact (e.g., skills were built, but not the confidence, to use the intervention).

Understanding relevant implementation mechanisms and their associated preconditions, mediators, and moderators is challenged by the complexity of health care systems [5]. Complex systems have numerous components that dynamically change over time, exhibiting behaviors that influence an implementation strategy’s success. For instance, in the training implementation strategy example above, group training sessions instead of one-on-one sessions may increase or decrease clinicians’ desire to learn through a mechanism such as social learning. If these changes are prevalent among the clinicians, then the strategy’s impact may be increasingly enhanced or diminished. The strength of this impact may also attenuate or non-linearly vary over time. Systems analysis methods that specialize in characterizing, modeling, and managing complexity are needed to grow knowledge regarding implementation phenomena within these complex systems (i.e., knowledge that under certain conditions, a strategy will be operated through one or more specific mechanisms to affect one or more implementation outcomes).

Specifically, whether an implementation strategy activates a mechanism within a system may depend heavily on dynamically changing system components and their interconnections. The ability to make these complexities explicit is indispensable to identifying both the mechanism and the conditions that activate it, such that future implementation efforts seeking to target the mechanism can appropriately devise strategies that enable those conditions across different contexts. Systems analysis methods offer the very tools for unpacking such complexities of real-world systems in which mechanisms operate [6], as they are uniquely capable of monitoring interconnections within systems that dynamically change over time. These dynamics occur from non-linear changes and interconnected elements that lead to emergent phenomena such as policy resistance [7].

Systems analysis methods also allow for simulating observed and anticipated trends given a system’s dynamic complexity (e.g., emergent phenomena, structural changes resulting from implementation, feedback loops represented by changes in variables that result from endogenous changes within the system [8]). Furthermore, simulations can be calibrated with historical data to increase confidence in the model’s simulated outcomes for unobserved time periods (i.e., the future). These simulations can then be used to conduct experiments to explore questions such as anticipated system effects from an implementation strategy given contextual determinants (e.g., organizational size, structure of social networks), time points upon which changes of a given magnitude are expected to be observed (for multiple variables along the hypothesized causal pathway, including those within key feedback loops), and trade-offs such as who will benefit most from a given implementation approach [6, 7, 9, 10]. Such experiments are particularly valuable since it is infeasible to directly test (e.g., through a randomized controlled trial) each of the many potentially relevant conditions' (and their combinations') influences on mechanisms. Especially in support of implementation science’s mission to accelerate real-world impact, systems analysis methods can complement existing interventional and observational methods to more comprehensively model and iteratively refine our understanding of implementation mechanisms.

## Methods

Systems analysis methods are approaches offered by the fields of systems engineering and systems science that apply qualitative or quantitative modeling techniques to reflect complexity within a system and identify optimal solutions given the system’s context. These methods focus on identifying and evaluating properties of complex systems (such as interactions between heterogeneous system components, feedback loops, dynamic relationships, and emergent behaviors resulting from heterogeneous, adaptive actors), thereby demystifying the relationships between a system’s components and changes to the system over time. By making system boundaries and goals explicit, systems analysis methods may help minimize implementation resistance. Many aspects of systems analysis methods reside under the umbrellas of systems science and systems engineering, which aim to grow knowledge regarding systems-related phenomena and to develop specific solutions to problems faced by complex systems, respectively [11]. Applied to studying implementation mechanisms, systems analysis methods can help (i) better identify and manage conditions that may or may not activate mechanisms (both expected mechanisms targeted by a strategy and unexpected mechanisms that the methods help detect) and (ii) flexibly guide strategy adaptations to address emergent influences of context (e.g., individuals’ motivations, norms, organizational policies and structures, financial resources) on the mechanisms that were not foreseen when the strategy was initially selected and used.

Wagner and colleagues [12] define the systems engineering approach to studying health systems as “an approach that uses data to improve decision-making … by (a) diagnosing problems and identifying needs, (b) evaluating decision options to address a selected problem or need through modeling or optimization, and (c) translating optimized decision options into practical recommendations or actions.” Building on this definition, we outline four steps to apply systems analysis methods for studying implementation mechanisms. To illustrate the steps, we use Lewis and colleagues’ depression screening implementation [4] as a case example and point the reader to other published literature relevant to the steps. We conclude by discussing the steps’ strengths, limitations, and implications for future implementation mechanisms research. Additional file 1 provides the Strengthening the Reporting of Observational Studies in Epidemiology (STROBE) guidelines [13] that we consulted in reporting our work (since we drew on published works to demonstrate the application of systems analysis methods to studying implementation mechanisms, without interventional attempts to impact the methods’ applications).

## Results

### Steps to apply systems analysis methods for studying implementation mechanisms

**Fig. 1:**
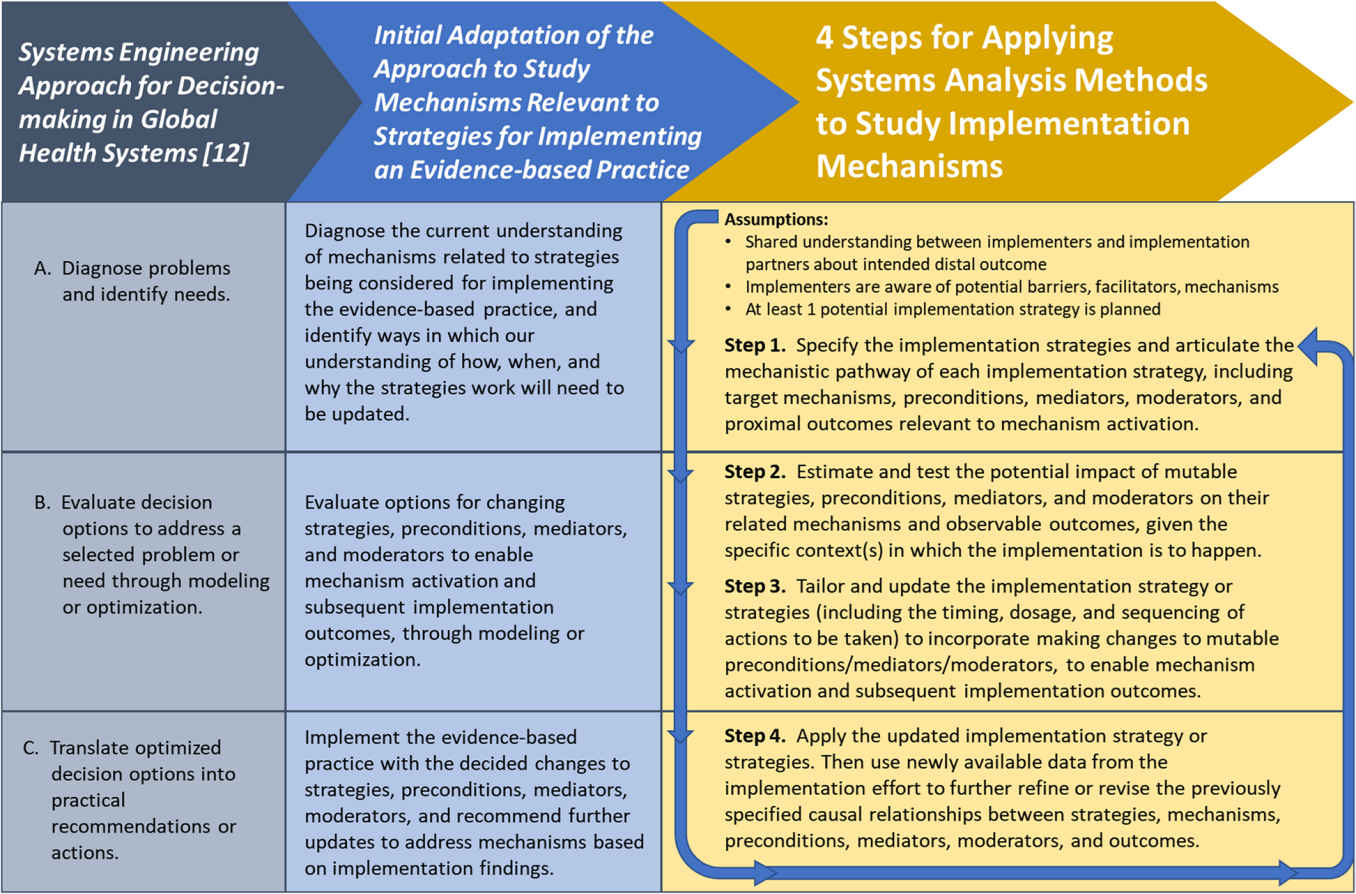
Evolution of the systems engineering approach [12] into four steps for applying systems analysis methods to study implementation mechanisms

Figure 1 shows how we extended Wagner and colleagues’ work [12] to arrive at our steps for applying systems analysis methods to study implementation mechanisms. For our steps, we start from the point at which an active implementation effort has yet to be launched. During this pre-implementation phase, we assume that:

- There is a shared understanding between implementers and their implementation partners about the intended distal outcome.
- Implementers are aware of at least some of the key potential barriers, enablers, and relevant mechanisms.
- At least one potential implementation strategy that accounts for these barriers, enablers, and mechanisms is planned for use in achieving the intended distal outcome.

The “Discussion” section elaborates on ways in which systems analysis methods can guide implementation endeavors prior to this starting point—e.g., deciding whether to launch an implementation effort at all, selecting an innovation to implement, or identifying potential barriers, enablers, and mechanisms to accordingly inform new strategies.

We chose the Lewis et al. [4] case example to help advance the field’s understanding of mechanisms by promoting cohesiveness across mechanisms-related papers. In this example, a community mental health center aims to implement a brief depression symptom severity screening measure (Patient Health Questionnaire). The distal outcome targeted is enhanced depression screening fidelity. We refer to two of the implementation strategies considered by the implementers: (i) Patient Health Questionnaire administration training and (ii) financial disincentive for each missed screening opportunity. See Fig. 2 for an adapted visualization of the example, which we further explain under step 1.

**F﻿ig. 2:**
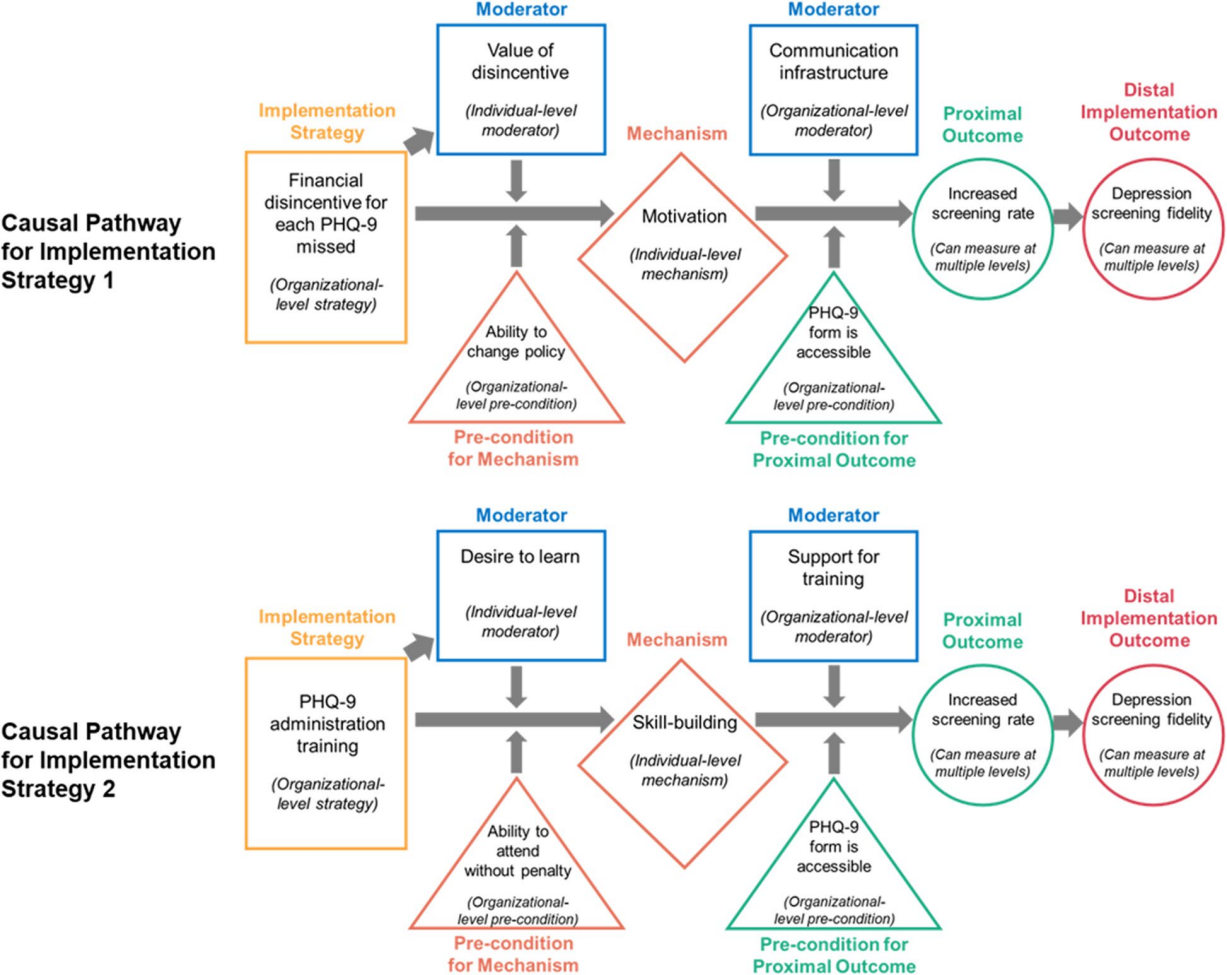
Example causal pathway diagrams, adapted from [4] Note: individual-level moderators and mechanisms can have group- and organizational-level implications; similarly, organizational-level moderators and preconditions can have individual-level implications

#### Step 1: Specify the implementation strategies and articulate the mechanistic pathway of each implementation strategy, including target mechanisms, preconditions, mediators, moderators, and proximal outcomes relevant to mechanism activation

Step 1 consists of four sub-steps that focus on specifying the (i) implementation strategy or strategies to apply, (ii) mechanisms expected to be at play through each strategy, (iii) preconditions/mediators/moderators that are expected to be relevant for mechanism activation, and (iv) relevant proximal outcomes that are expected to indicate mechanism activation. It is also important to specify these for multiple levels of the health care system [14]—e.g., clinician- versus organization-level moderators. For these specifications, one possible approach outlined by Lewis and colleagues [4] is to use causal pathway diagrams to specify the hypothesized relationships between strategies, mechanisms, preconditions, mediators, moderators, and outcomes.

Potentially useful systems analysis methods for step 1 include techniques that are widely used for quality improvement and patient safety, such as 5 whys, fishbone diagrams, and other tools used for root cause analysis [15, 16] that can be adapted to identify the causes for a mechanism being or not being activated. End users’ (i.e., implementation actors’) input is often used when conducting root cause analysis or failure modes and effects analysis [15–17]. For example, possible causes of the skill-building mechanism not being activated can be explored by using these structured root cause analysis tools to seek input from and consensus among implementation actors most knowledgeable about or experienced in skill-building for depression screening, as well as individuals that the mechanism involves (in this case, clinicians). Similarly, tools used for failure modes and effects analysis [17] can also be adapted to identify possible influences on the path from strategy through mechanism to outcome.

Causal loop diagrams (CLDs) are another useful systems analytic tool for identifying how mechanisms relate to the selected implementation strategies and targeted implementation outcomes [7]. Although CLDs, like causal pathway diagrams, focus on how specific factors (e.g., preconditions) interconnect to cause changes in outcomes, CLDs emphasize dynamic change. CLDs characterize how factors or behaviors emerge and perpetuate over time through consistently escalating or de-escalating trajectories (reinforcing feedback loops). CLDs also illustrate how the introduction of a particular variable helps a system reach or maintain equilibrium (balancing feedback loops). {Note: Feedback loops have unique definitions across related, but distinct, fields such as psychology [18–21]. For the purposes of this paper, we follow the definitions used in systems engineering and system dynamics, in which a “balancing feedback loop” is made up of interrelated factors that together lead to a system returning to the status quo or reaching a type of equilibrium due to goal-seeking behaviors or system constraints that prevent perpetual improvement or perpetual decline (i.e., reinforcing feedback loops) [7, 9, 10, 22, 23]. For example, even with the most successful implementation strategy to reduce clinic wait times through structural efficiencies (e.g., administration), the rate at which individuals leave the wait list will always be restricted (or “balanced”) by the number of clinicians and the average client’s time in care [24, 25]. The following description of Fig. 3 provides additional examples of balancing feedback loops.} Figure 3 depicts a CLD of the example causal pathway diagram components, from which several insights emerge:

1. The CLD makes it clearer how central organizational leadership supports the use of the two implementation strategies and their preconditions. Furthermore, the strategies’ success is hypothesized to increase leadership support over time, increasing the likelihood of implementation sustainment. Thus, CLDs can help identify measurement targets—in this case, the CLD suggests that measuring organizational leadership over time can help monitor the likelihood of implementation success.
2. The CLD identifies two critical balancing loops, B1 and B2, that were unidentified in the causal pathway diagram.
  - B1 suggests that the level of incentive required to change motivation influences the value of a disincentive on motivation—a value which will likely vary by clinician based on factors such as their salary, time in position, and perceived burden of conducting the Patient Health Questionnaire-based screening.
  - B2 highlights how the hypothesized pathway by which a financial incentive increases screening might vary over time, namely, as fidelity increases and is easier to achieve (requiring a lower threshold to maintain clinician’s motivation to increase their screening rate), the financial incentive may be decreased.

- S﻿uch insights from CLDs have important implications for planning the allocation of resources associated with an implementation strategy over time.

3. The CLD highlights a shared mechanism—skill building—that was previously only associated with one of the two strategies. Thus, CLDs are also a promising way to identify efficient bundles of implementation strategies that synergistically benefit from the activation of shared mechanisms.

**﻿Fig. 3:**
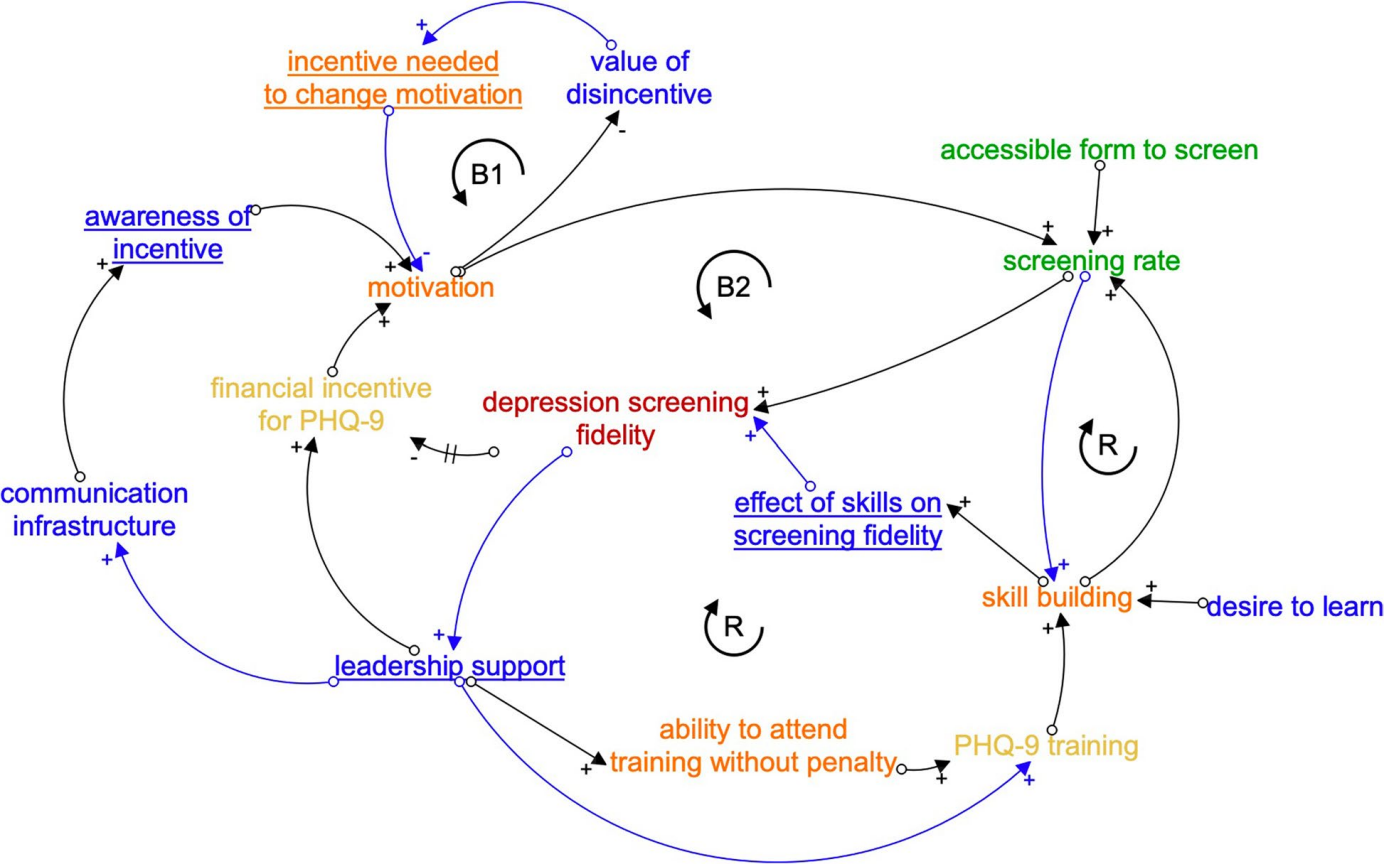
A causal loop diagram of the example causal pathway diagram components in Fig. 2; color scheme follows that of Fig. 2 Note: underlined are new variables that are not among the causal pathway diagram components in Fig. 2; black arrows indicate new pathways not depicted in Fig. 2

Causal pathway diagrams or similar diagrams generated in this step not only point to hypothesized relationships, but importantly also imply which relationships should not exist. For example, as diagrammed in Fig. 2, the “ability to attend training without penalty” precondition and the “clinician desire to learn” moderator are not related. If available data (e.g., from previous studies) show a relationship between preconditions and moderators that should not exist, or if the relationship identified does not reflect reality, it is an indication that the diagram, and in turn the understanding of the causal pathway, is incomplete and warrants an update. Comprehensive identification of such testable implications of a hypothesized causal pathway can rely on existing causal inference tools. For instance, the pathway can be expressed using a CLD as above or a directed acyclic graph (systematic visual representations of causal relationships) [26] to understand confounders and potential sources of bias in the pathway between strategy and outcome.

Scenario simulation can be helpful for updating the mechanistic pathway to be consistent with existing data. For implementation efforts more generally (not specific to step 1), simulations allow implementers to computationally “try out” different scenarios of implementation strategies and observe their potential impact on outcomes, prior to deploying the strategies. Multiple scenarios can be simulated to estimate different means and ranges of outcomes arising from incomplete data/knowledge or to identify implementation strategies that seem most robust to the uncertainty even under limited resources (i.e., a mathematical optimization problem of finding the best solution given constraints—e.g., multiphase optimization strategy [27]). Because simulation models do not rely only on already available or easily measurable data, they can be used to identify what data are needed for more precise outcome estimates or strategy design, and how much of a difference having additional data would make. As explained by Sheldrick and colleagues [24], simulation models can thus be used to conduct virtual experiments that help decision-makers consider the trade-offs in using a given implementation strategy over another. (Sheldrick and colleagues recommend using simple models, such as Monte Carlo models, that are transparent and easily understood by decision-makers with limited time to engage with the models. Alternatively, more complex system dynamics, agent-based, and microsimulation models can also support decision-makers’ learning with appropriate, acceptable guidance [25, 28, 29]).

A range of potential causal pathways describing the mechanisms can be simulated to identify ones that best match available knowledge and data. As per Fig. 2, if available data show a relationship between the “ability to attend training without penalty” precondition and the “clinician desire to learn” moderator, thereby contradicting the currently conceptualized causal pathway, then alternative relationships can be “tried out” using the simulation model. For instance, a direct relationship between the two components, or a relationship that connects through a third (either specified or unspecified) component, can be built into alternative versions of the model. After enumerating all such feasible causal pathways, it is possible to choose one that most closely matches the available data—i.e., select a model version that minimizes the difference between available data and model-simulated data. If the selected model has one or more unspecified components, it means that mechanisms may be at play that were not originally hypothesized; this warrants an update to the conceptualized causal pathway before proceeding. The unspecified components’ placement in the model can help researchers speculate about missing mechanisms and/or what to measure and when to identify those mechanisms.

#### Step 2: Estimate and test the potential impact of mutable strategies, preconditions, mediators, and moderators on their related mechanisms and observable outcomes, given the specific context(s) in which the implementation is to happen

Using the simulation model of the causal relationships between strategies, mechanisms, preconditions, mediators, moderators, and outcomes, we can virtually test the impact of changing different combinations of the mutable strategies, preconditions, mediators, and moderators in the model. Mutable components are ones that we consider to be realistically changeable as a part of the implementation effort. For instance, the “Patient Health Questionnaire form is available/accessible” precondition may be mutable for implementation settings in which the form can be made available to clinicians through an existing electronic health record system. We can use simulation to estimate the relative impact of these changes on whether and how hypothesized mechanisms are activated and how outcomes are subsequently affected.

Simulations can also help set realistic expectations for mechanism activation and/or the strength of a mechanism’s influence on implementation outcomes. For instance, we can simulate whether there are threshold levels or tipping points of the precondition “Patient Health Questionnaire form is available/accessible” and the moderator “organizational support for training” at which changes in their values do not meaningfully change their impact on the increased screening rate. Such an insight would help make valuable decisions about resource efficiency (i.e., not devoting more time and resources beyond these thresholds). If such thresholds are simulated, the implementation effort can accordingly monitor when threshold levels are reached and resources can be reallocated toward other mutable strategies, preconditions, mediators, and moderators to further activate targeted mechanisms and drive desirable outcomes.

Importantly, simulations can help identify measurement targets along the modeled causal pathway that are more or less sensitive at detecting mechanism activation. For instance, if simulated scenarios for the depression screening example indicate very small changes in the increased screening rate despite large changes in the level of skill building, then a more immediate indicator than the screening rate is needed to specifically gauge the extent to which the skill-building mechanism is activated (e.g., the number of clinicians whose skills are built through training). If feasible, the implementation effort can accordingly plan to monitor this more immediate indicator to measure mechanism activation more accurately given the expected timeline and magnitude of change.

#### Step 3: Tailor and update the implementation strategy or strategies (including the timing, dosage, and sequencing of actions to be taken) to incorporate making changes to mutable preconditions/mediators/moderators, to enable mechanism activation and subsequent implementation outcomes

Model simulations as described under step 2 can be used to select and shape the strategy or a combination of strategies for implementation. For instance, the relative impact of using one or both of the “Patient Health Questionnaire administration training” and “financial disincentive for each missed screening opportunity” strategies can be simulated under varying preconditions/mediators/moderators to decide whether one or both strategies should be used simultaneously or sequentially, and with the same or different types of implementation actors (e.g., executive director, clinician, front-line staff, patients across heterogeneous contexts), to reach desired implementation outcomes. Similarly, if moving forward with both strategies, simulation can help determine which and how many resources to allocate toward enhancing the “organizational support for training” precondition versus the “organizational communication infrastructure” precondition for optimal impact.

As described in step 1, enumerating and selecting a strategy or a combination of strategies to use among all realistic options can be posed as an optimization problem. Namely, using a simulation model, all potential scenarios of utilizing one or more strategies that trigger changes in one or more mutable preconditions/mediators/moderators can be simulated to identify the scenario(s) expected to maximize desirable outcomes while minimizing the effort (or some other optimization factors, such as resources) needed to reach those outcomes. The implementation strategy or strategies can subsequently be tailored and updated to mirror the identified optimal scenario(s). Especially as multi-strategy implementation efforts have become the norm for promoting the uptake of evidence-based practices, simulations can inform how multiple potential strategies can be combined to enable mechanism activation given an implementation setting’s key contextual factors that include relevant preconditions, mediators, and moderators. Importantly, simulations can help predict multiple strategies’ combined effects on mechanisms that, given system complexities, may not be simply additive in nature and thus difficult to predict using other methods.

#### Step 4: Apply the updated implementation strategy or strategies, then use newly available data from the implementation effort to further refine or revise the previously specified causal relationships between strategies, mechanisms, preconditions, mediators, moderators, and outcomes

We can now apply the implementation strategy or strategies devised in step 3 and assess implementation outcomes. During this step, it is necessary to collect data on components in the causal pathway that were identified in step 2 as most indicative of mechanism activation (e.g., the number of clinicians whose skills are built through training, as mentioned above). To grow our understanding of implementation mechanisms, it is critical to then reconduct step 1 with the newly available data; in other words, we must use the new data to test whether the implications of the hypothesized causal relationships still hold (e.g., whether the “ability to attend training without penalty” precondition and the “clinician desire to learn” moderator remain unrelated, as mentioned above). If the new data contradict the currently conceptualized causal pathway, then tasks outlined under step 1 can be followed to explore alternative relationships that better explain both previous and new data (examples of this are outlined in the “Examples of systems analysis methods for studying implementation mechanisms” section) and to accordingly update the pathway.

### Examples of systems analysis methods for studying implementation mechanisms

Various systems analysis methods can be used for studying implementation mechanisms, not limited to the ones that are mentioned in the steps’ descriptions in the “Steps to apply systems analysis methods for studying implementation mechanisms” section above. Table 2 shares examples from the literature of systems analysis methods that are beginning to be used for mechanisms research, curated using the approach described below. Recognizing that the different methods may also vary widely in the level of systems analytic expertise needed for their use, the table also refers the reader to tutorials and other resources that can help them decide whether they need to engage collaborators with additional expertise to pursue using a listed method.

**Table 2:** Published examples of systems analysis methods being used to study implementation mechanisms

Systems analysis method	Definition	Usage example	References for more information
Agent-based modeling	Computational modeling that simulates actions and interactions of actors (e.g., individuals, groups, and/or organizations) within a system, as well as system behavior and outcomes emerging from the interactions of these actors and each actor’s assigned characteristics [30]	Testing the hypothesis that actors strongly identifying with their organizations is a mechanism that drives intraorganizational behavioral divergence and network polarization for the implementation of evidence-based practices [31]	[32, 33]
Causal pathway diagramming	Visual representation of causal, dynamic interrelations among variables and outcomes of interest in a given context [4]	Specifying the mechanisms of action of implementation strategies used to improve acute stroke treatment practices [34]	[35, 36]
Participatory system dynamics	Use of a participatory epistemology to develop and integrate modeling of complex systems to support decisions that are currently least supported and to empower those who are typically the least empowered; models therefore are developed, reviewed, and acted upon in partnership with individuals who are affecting and affected by the system being modeled [37]	Determining the mechanisms through which past actions taken either enabled or constrained clinical guidelines implementation [38]	[25, 39]
Process evaluation	Assessment and documentation of the developed, planned, and delivered implementation strategy [40]	Ascertaining what characteristics of front-line staff and leadership served as mechanisms for hand hygiene improvement [41]	[42–44]
Realist evaluation	Generation of a theory-driven and practice-informed model that connects context, mechanisms, and outcome patterns [45]	Studying contextual factors and causal mechanisms that enabled or hindered evidence-based interventions’ sustainability [46]	[47, 48]
Ripple effects mapping	Participatory evaluation that generates a visualization of causes and their cascaded effects radiating from each cause, along with participants’ first-hand experiences [49]	Establishing context-mechanism-outcome configurations based on mixed-methods data for an intervention to increase physical activity [50]	[51, 52]
Simulation modeling	Creation and analysis of a computational model of a system to replicate and anticipate the system’s real-world behavior [53]; of note, some of the other listed methods (e.g., agent-based modeling and participatory system dynamics) are subsumed under this method	Articulating and refining implementation actors’ articulation of hypotheses regarding causal mechanisms of the intervention and implementation strategies [24]	[29]
Structural equation modeling	Mathematical representation of a phenomenon to investigate aspects of the phenomenon that are statistically and/or causally related to one another and to the phenomenon [54]	Examining the mediators of hospitals’ escalation prevention potential as mechanisms for successful electronic health record implementation [55]	[56–58]

We conducted a targeted search of the literature and ongoing studies to identify key examples of systems analysis methods applied to elucidating and testing mechanisms. We searched PubMed and NIH Reporter and used forward searching for a select set of seminal articles. Within PubMed, we crafted the following search string, based on a modified version of the search string utilized by Lewis and colleagues [59] for implementation science and mechanisms research, and the specific systems analysis methods identified by Wagner, Crocker, and colleagues [12]:

((Implement*[tiab] OR disseminate*[tiab] OR “knowledge translation”[tiab]) AND (Mediate*[tiab] OR moderator[tiab] OR mechanism*[tiab]) AND (“empirically supported treatment”[tiab] OR “evidence-based practice”[tiab] OR “evidence-based treatment”[tiab] OR innovation[tiab] OR guideline[tiab]) AND (“structural equation model*”[tiab] OR “directed acyclic graph”[tiab] OR “DAG”[tiab] OR “causal loop diagram”[tiab] OR “process evaluation”[tiab] OR “process analysis”[tiab] OR “optimiz*”[tiab] OR “simulat*”[tiab] OR “agent”[tiab] OR “quality improvement”[tiab] OR “fish bone”[tiab] OR “failure modes and effects analysis”[tiab] OR “FMEA”[tiab] OR “Ishikawa”[tiab] OR “flow map*”[tiab] OR “process map*”[tiab] OR “value stream map*”[tiab] OR “root cause analysis”[tiab] OR “PDSA”[tiab] OR “system dynamic*”[tiab])) NOT (Biology OR microbiology)

The search string that we used in PubMed (shown above) covered synonyms and variations of implementation science, evidence-based interventions, and implementation mechanisms, as well as various systems analysis methods, while attempting to exclude works that discuss biological mechanisms. Then, within NIH Reporter, we crafted the following two search strings, which were limited by the search capabilities of the database: (1) “mechanism” AND “implementation science” and (2) “mechanism” AND “implementation research.” Finally, we forward searched two seminal papers ([4] and [59]) within Google Scholar.

The search was conducted during March and April 2022. Each entry identified by the search strategies was reviewed by one member of the authorship team for relevance. After the first ten entries identified by each search strategy were screened, the authorship team met and discussed the relevance of the articles being returned, agreeing by consensus to proceed with the search strategy and complete the full screening. Those entries deemed to be relevant examples of systems analysis methods applied to elucidating and testing mechanisms were then included; relevant fields were extracted by one member of the authorship team using a pre-set template, including the fields in Table 2, then reviewed and refined by all members.

The examples that we found of systems analysis methods applied to studying mechanisms ranged from primarily quantitative (e.g., structural equation modeling [45]) to primarily qualitative (e.g., realist evaluation [36]) approaches. Visual representations of mechanisms and additional interrelated factors that influence implementation were central to several of the examples (e.g., causal pathway diagramming [25], ripple effects mapping [40]), while other examples focused on the computational modeling of those interrelationships (e.g., agent-based modeling [22], simulation modeling [16]). Emphasized throughout the examples (particularly process evaluation [30] and participatory system dynamics [28]) was the intentional, close incorporation of the perspectives of individuals involved in the modeled system or systems in which implementation was occurring. These individuals were involved in identifying which system components to model, defining model boundaries, proposing relevant mechanisms and their connections to contextual factors, and interpreting the relevance of model outputs for implementation efforts.

It is worth noting that the examples shown in Table 2 use systems analysis methods predominantly for steps 1 and 2—i.e., for identifying potential mechanisms and for assessing the expected impact of strategies and contextual factors on mechanisms. Even as implementation research generally may be embracing the use of systems analysis methods more, our search had difficulty finding studies that use systems analysis methods for steps 3 and 4—i.e., for refining strategies to explicitly enable better mechanism activation and for examining the resulting changes on mechanism activation.

## Discussion

We provide a four-step approach to applying systems analysis methods for examining implementation mechanisms. The steps integrate Wagner and colleagues’ systems engineering approach for decision-making in global health systems [12] with Lewis and colleagues’ approach to identifying and studying mechanisms of implementation strategies [4], to guide the practical use of systems analysis methods for mechanisms research in implementation science. To demonstrate the steps, we use as a case example Lewis and colleagues’ implementation of the Patient Health Questionnaire for depression screening in a community mental health center [4]. We also point the reader to additional examples of systems analysis methods and resources that can guide their usage in future implementation research.

### Systems analysis methods’ roles in making mechanism-related assumptions explicit

The steps encourage rigorous specification and methodical refinement of assumptions surrounding the mechanisms that are expected to be at play when implementation strategies lead to the uptake of evidence-based interventions into routine practice. These assumptions relate to which implementation strategies target specific mechanisms, and the preconditions, mediators, and moderators specific to implementation contexts that influence a strategy’s ability to activate mechanism(s) necessary for implementation success. Unless these assumptions can be specified, the reason why an implementation strategy is or is not successful cannot be fully understood. Without this understanding, it cannot be made explicit how the strategy can be improved for continued use or tailored to fit new implementation contexts.

### Considerations for multi-level systems

Systems analysis methods are used in a variety of disciplines to understand how components within and across systems change and interact with one another to affect system properties. Especially for multi-level systems common in health care and community settings—for which system properties (e.g., implementation outcomes, moderators) are affected by components at multiple levels (e.g., individuals, clinics, organizations, community)—making changes to the system requires strategies (e.g., implementation strategies) that target multiple levels (e.g., training for clinicians, update to organizational policy) and key leverage points (i.e., factors in a system that drive change) [10]. As strategies become multi-level and complex, so do the causal pathways that link strategies to mechanisms to outcomes. Techniques involving structured inquiries (e.g., root cause analysis), visualization (e.g., causal pathway diagrams), computational modeling (e.g., scenario simulation), and other systems analysis methods can help accurately characterize and manage the complex knowledge regarding multi-level system components and their interrelationships. In the setting of multiple strategies, systems analysis methods can capture and reflect synergistic, antagonistic, or other non-additive patterns of co-occurrence.

### Incorporating implementation actors’ input and conceptual guidance

Hypothesized causal pathways, from implementation strategies through mechanisms to outcomes, should stem not only from theories and frameworks, but also from the experiences, values, and beliefs of implementation actors [60]—individuals who impact and/or are impacted by an implementation effort. It is thus important to leverage actor-engaged approaches that capture multiple perspectives to complement, or officially be a part of, efforts to apply systems analysis methods to mechanisms research. For instance, actors’ mental models of how different factors are linked to outcomes are critical to accurately characterizing and building the system structure underlying a computer simulation model to be used in examining potential scenarios [9, 61, 62]. Hypothesized strategy-mechanism-outcome links may also be based on one or more theories, models, and frameworks that categorize or provide explanations for implementation-related phenomena. Reviewing the domains and their relationships per an implementation-relevant theory/model/framework {e.g., Exploration, Preparation, Implementation, Sustainment (EPIS) framework [63]} can prompt consideration of mechanisms and causal pathways that have not previously been considered (e.g., EPIS’ bridging factors that span multi-level outer and inner contexts [64, 65]), which can be specified and examined using systems analysis methods. Such examinations may also have opportunities to reciprocally inform implementation theories/models/frameworks of the relative prevalence, strengths, and further specifications of their domains and relationships as observed through implementation mechanisms research.

### Leveraging concurrent advances in other aspects of mechanisms research

For such actor-engaged and theory/model/framework-aligned examination of implementation mechanisms to successfully apply systems analysis methods (i.e., carry out the four steps outlined in this article), concurrent advances in other aspects of mechanisms research are indispensable. The steps, and particularly whether an iteration back to an earlier step is warranted, depend on comparing the systems analysis method-based observations (e.g., simulation results) to available empirical implementation knowledge and data. Especially as the observations suggest measures that implementation efforts can focus on collecting (e.g., for better indication of mechanism activation), the suggested measurements can only yield useful data if measurement approaches are and continue to be practical, accurate, timely, and replicable. Relatedly, to iteratively refine understandings of causal pathways from implementation strategies to outcomes across one or more implementation efforts with shared target mechanisms, it is important to have methods for clearly documenting when and how specific mechanisms are tracked and examined, as well as methods for tracking and evaluating resulting implementation and clinical outcomes or other observations. Visualizations of causal pathways (e.g., CLDs), simulation records (e.g., simulated/computed model outputs), and other documentations (e.g., fishbone diagrams) generated from applying systems analysis methods to implementation mechanisms research can offer some approaches to documentation, while a wider consensus across the field is necessary for shared terminologies and other conventions for documentation [66].

### Limitations

Using systems analysis methods, such as those in the four steps described above, can help study mechanisms as they relate to multi-level strategies and contextual factors, require mechanism-related assumptions to be specified and tested, and identify targets along the causal pathway to inform mechanisms research. This work also has limitations. First, the starting point for the steps assumes that the decision to implement, the distal outcome, and at least one implementation strategy are set. Although this starting point allows the iterative nature of the steps to be underscored, it leads to excluding explicit discussion within this article of the potential utility of systems analysis methods for pre-implementation efforts to decide whether to implement, agree with implementation actors (particularly those who might be end users of a simulation model or with implementation decision-making authority) on the distal outcome, and inform the selection of an initial strategy. We encourage readers to refer to implementation mapping [67], group model building [68], and other established implementation research methods that focus on problem identification, implementation needs assessments, outcomes selection, and strategy design. Second, we use a single case example of depression screening implementation to outline the steps to apply systems analysis methods for studying implementation mechanisms. While implementation efforts concerning different evidence-based practices and settings may call for considerations distinct from that of our case example, we aligned to established case study research practices of focusing on a single case when the case is atypical and noteworthy [53, 54]. We therefore chose to anchor the illustration of our ideas on this example that is unique in its association with a seminal paper that both conceptualizes mechanisms of implementation strategies and establishes a visual representation of foundational mechanisms-related definitions [4] on which we build in this manuscript. Third, given that this article is the first in articulating explicit steps for applying systems analysis methods for implementation mechanisms research, neither the main case example that we use to illustrate the steps nor the examples that we point to in the literature for each step were pursued by their implementation team with these specific steps in mind. Although this work is grounded in both a review of systems-based health research methods [12] and foundational definitions and examples of mechanisms-related concepts [4], further work is needed to prospectively apply and test these now articulated steps for mechanisms research.

## Conclusions

Especially as implementations of evidence-based interventions increasingly target underserved populations and are pursued in low-resource settings that contextually differ from the high-resource settings in which the interventions were originally developed and implemented, elucidating the mechanisms that explain the “why” and “how” of implementation is more essential now than ever before [69, 70]. Systems analysis methods, widely used in multiple disciplines to investigate causal relationships and behaviors of complex systems, offer opportunities to identify, specify, test, and refine our understanding of implementation mechanisms that need to be activated for implementation strategies to lead to desirable outcomes. We hope that the four steps to applying systems analysis methods we introduced here can encourage more mechanisms research efforts to consider these methods and in turn fuel both (i) rigorous comparisons of these methods to alternative mechanisms research approaches and (ii) an active discourse across the field to better delineate when these methods are more or less appropriate to use for advancing the knowledge regarding implementation mechanisms.

### Supplementary Information

**Additional file 1. **STROBE Statement—checklist of items that should be included in reports of observational studies.

## Data Availability

No new data were used in this work. Information supporting the work’s claims is available within the article.

## Abbreviations

CLD: Causal loop diagram
EPIS: Exploration, Preparation, Implementation, Sustainment
NIH: National Institutes of Health
PHQ-9: Patient Health Questionnaire-9
STROBE: Strengthening the Reporting of Observational Studies in Epidemiology
